# Book Review: A Book Review on Roma Minority Youth Across Cultural Contexts Taking a Positive Approach to Research, Policy and Practice

**DOI:** 10.3389/fsoc.2021.809712

**Published:** 2022-01-14

**Authors:** Umit Ozturk

**Affiliations:** Freelance Academic, Ph.D. in Sociology, Diyarbakır, Turkey

**Keywords:** roma minority, roma youth, positive youth development (PYD), race and ethnic (minority) issues, multicularism

**A Book Review on**

**Roma Minority Youth Across Cultural Contexts Taking a Positive Approach to Research, Policy, and Practice**

by Radosveta Dimitrova, David Sam and Laura Ferrer-Wreder (2021). Oxford, United Kingdom: Oxford University Press, 2021, 264 pages, ISBN: 9780190654061 https://global.oup.com/academic/product/roma-minority-youth-across-cultural-contexts-9780190654061?q=dimitrova&lang=en&cc=us#

This Oxford University Press volume edited by Radosveta Dimitrova, David Sam and Laura Ferrer-Wreder is a valuable resource for global multidisciplinary audience and the readership of the Frontiers Sociology Section on Race and Ethnicity. The book applies a strength based multidisciplinary and interdisciplinary approach to human development by investigating the most stigmatized and marginalized ethnic minority in Europe from a global cultural perspective. The main focus is on Positive Youth Development (PYD) approaches among Roma minority youth across diverse societies uncovering interconnectedness between nation states, ethnic, national, racial and religious identities and contexts as powerful facets in demarcating social, political and economic boundaries structuring life chances for Roma populations. The book discovers shared socio-cultural world through participatory and broader collective processes that indicate how Roma are experiencing these social processes. In so doing, the volume capitalizes on multiple assets within the collective potential of Roma communities globally embedded in historically intertwined racial and ethnic conflicts, discrimination, segregation and marginalization that regrettably are still vivid reality for Roma minority groups in many Eastern European and Western countries. By drawing on such theoretical backgrounds, the aim is to provide an international and interdisciplinary forum for scholarly investigations and discussions that will advance our basic knowledge of marginalized minority groups in its historical and socio-cultural contexts. The orientation of the volume is towards formulating new conceptualizations of optimal development embedded in culture, race and ethnicity, together with theoretically relevant empirical investigations and conceptual contributions.

The volume incorporates a stellar cast of contributions by leading scholars in sociologically-centered fields, as well as developmental and cross-cultural psychology, Roma and ethnic minority studies, political science, social policy, health, education, social psychology, cultural studies, prevention, intervention, policy and practice fields with original empirical contributions and conceptual analyses on relevant aspects of race, ethnicity, culture and religion among Roma populations. Such contributions successfully integrate multidisciplinary and interdisciplinary work that provides connections between different sub-fields within the broader umbrella of PYD conceptual orientation. Therefore, by building on PYD theoretical and methodological foundations, the volume advances and informs future research, policy and practice in addressing racial and ethnic inequalities faced by Roma populations globally.

The volume opens with a foreword by one of the founders of PYD, Prof. Richard M. Lerner and comprises three parts for a total of 13 chapters. Part A regards the historical overview of major socio-demographic, cultural and contextual characteristics of Roma populations across different settings. Part B presents theories on adaptation on PYD traditional frameworks supplemented by Part C that offers novel empirical findings on PYD and well-being of Roma in a number of regions across Europe and United States. Each contribution pays special attention to cultural specifics and universals of the findings to promoting optimal development, health and well-being among Roma and in other similarly oppressed ethnic minority groups.

Confronting racism, classism, stigma, marginalization and similar discriminations within the Roma context requires an uncomfortable recognition of unequal privileges that these young people counter in their life. Authors expand on these challenges through thoughtful narratives and empirical findings that highlight questions regarding social position and class, provision of early childhood services, actualizing change with Roma youth and their communities, social connectedness, education, identity and how different identities are intertwined in a global self-concept, how positive development framework can improve lives of excluded people and relevant strategies for dealing with challenges as they emerge. Following this line of thought, contributors share diverse and multifaceted approaches to promoting equitable society, resources and improve well-being and quality of life among marginalized minority groups. Throughout the volume, policy recommendations are offered to the readers with suggestions on improving life conditions of the next generation of Roma in a global perspective.

For sociologists and interested scholars looking for ways to utilize positive development frameworks within own work, an engaging chapter was on “Reframing the Narrative: Revealing Positive Youth Development in the Self-Descriptions of Roma Adolescents.” In this chapter, the authors - Johnson, Thelamour, Sankar and Dimitrova - present self-reflective analyses of how multiple identities interact with Roma youth perceptions and how keeping those interaction effects in mind lead to specific self-views. The chapter examines self-descriptors of Roma adolescents by quantitatively exploring the relations of self-esteem, ethnic identity, and self-description domains of future orientations, relationships, and personal characteristics. The results showed less critical self-references among youth with high self-esteem (e.g., “I am joyful and much in love”) in that despite experiences of isolation and negative self-evaluation, self-descriptors indicated a deep sense of family closeness and meaningful friendships with peers (e.g., “In my life, I value my family and real and good friends”). The chapter argues for the existence of positive self-systems among Roma youth despite complex life challenges.

This volume is an accessible guide for scholars and educators dedicated to teaching and learning within the framework of positive development, social justice and interdisciplinary connections of modern social sciences that place cultural and socio-cultural interaction on a center stage, and with an eye to the current socio-cultural climate in Europe and the United States. Though written with a multidisciplinary audience in mind, the volume is well-situated to offer practical tips and best practices that address the needs of marginalized groups and scholars and stakeholders who are committed to tackling social exclusion and marginalization and digging deeper into issues surrounding race, social class, inequality and ethnicity. The volume may also be an inspiration for graduate students and recent PhDs and instructors who believe this area of study allows for such inquiries in ways that address difficult subjects that highlight social inequities. The volume is relevant read for scholars from a variety of disciplines such as sociology, anthropology, education, ethnography, cultural history, linguistics, psychology, social work, youth studies that will further enhance interdisciplinary commitment to the study of marginalized ethnic minority populations globally.

